# Efficacy and safety of darolutamide *versus* abiraterone acetate plus prednisone in combination with ADT for mHSPC: a real-world clinical retrospective study

**DOI:** 10.3389/fphar.2025.1608339

**Published:** 2025-06-19

**Authors:** Ting Hu, Fang Zhou, Yang Zheng, Bohan Luo, Yongliang Zhang, Chengpeng Gu, Guopeng Wang, Jinze Zhang, Jingzhi Tian, Yu Nie, Yunlin Feng, Shangqing Ren, Wenjia Di, Dong Wang

**Affiliations:** ^1^ School of Medicine, University of Electronic Science and Technology of China, Chengdu, China; ^2^ Robotic Minimally Invasive Surgery Center, Sichuan Provincial People’s Hospital, School of Medicine, University of Electronic Science and Technology of China, Chengdu, China; ^3^ Organ Transplantation Center, Sichuan Provincial People’s Hospital, School of Medicine, University of Electronic Science and Technology of China, Chengdu, China; ^4^ Department of Information, Sichuan Provincial People’s Hospital, School of Medicine, University of Electronic Science and Technology of China, Chengdu, China; ^5^ Clinical Medical College, Southwest Medical University, Luzhou, China; ^6^ Department of Nephrology, Sichuan Provincial People’s Hospital, School of Medicine, University of Electronic Science and Technology of China, Chengdu, China

**Keywords:** metastatic hormone-sensitive prostate cancer, darolutamide, abiraterone acetate, androgen deprivation therapy, efficacy, safety

## Abstract

**Introduction:**

Effective treatment during the metastatic hormone-sensitive prostate cancer (mHSPC) stage is crucial for delaying disease progression. Due to the lack of a head-to-head comparison of darolutamide (DARO) and abiraterone acetate plus prednisone (AAP) doublet regimen, this study aims to compare the efficacy and safety of DARO + ADT and AAP + ADT in the treatment of mHSPC in the real world.

**Methods:**

This study retrospectively analyzed patients with mHSPC who received DARO or AAP treatment in Sichuan Provincial People’s Hospital from January 2022 to June 2024, with follow-up until December 2024. The clinical data and prostate-specific antigen (PSA) changes of patients were collected. The primary endpoint was time to metastatic castration-resistant prostate cancer (mCRPC), and the secondary endpoints were overall survival (OS), radiological progression-free survival (rPFS), time to PSA progression, time to pain progression, and time to subsequent prostate cancer therapy.

**Results:**

A total of 178 patients were included, with 96 in the DARO group and 82 in the AAP group. The baseline characteristics of the two groups were comparable. The median follow-up time and interquartile ranges of the DARO and AAP groups were 12.0 [7.9–17.6] months and 17.4 [9.3–23.8] months, respectively. For the primary endpoint, DARO significantly delayed the time to mCRPC *versus* AAP [HR, 0.41 (95%CI, 0.23 to 0.71); *P* < 0.005]. And the DARO group significantly benefited in all secondary endpoints. DARO significantly led to deeper PSA reduction compared to AAP, with higher median reduction rates, better PSA50 and PSA90 remission rates, and a higher proportion of patients reaching lower PSA values. The incidence of adverse reactions was similar in the two groups, and there was no grade 3 or above drug-related adverse reactions.

**Conclusion:**

In the treatment of mHSPC, DARO + ADT was associated with significant improvement of clinical outcomes *versus* AAP + ADT, while their safety is comparable.

## 1 Introduction

Prostate cancer (Pca) is a malignant tumor originating from the epithelial cells of the prostate gland (Rebello et al., 2021). Global cancer statistics indicate that prostate cancer is the second most common cancer in men and the fifth leading cause of cancer-related deaths among males (Smith et al., 2023). Metastatic hormone-sensitive prostate cancer (mHSPC) represents a critical stage, where timely and effective treatment can potentially delay disease progression to metastatic castration-resistant prostate cancer (mCRPC) and improve overall prognosis (Castellan et al., 2018; Chi et al., 2019; Armstrong et al., 2019).

Androgen deprivation therapy (ADT) has long been the cornerstone of treatment for mHSPC (Yu and Aragon-Ching, 2022). However, traditional approaches such as ADT alone or ADT combined with first-generation antiandrogens like bicalutamide have proven insufficient to significantly improve survival or quality of life in mHSPC patients (Ueda et al., 2024; Wenzel et al., 2023). Moreover, nearly all advanced prostate cancer patients eventually develop resistance to ADT and first-generation androgen receptor antagonists, leading to progression to mCRPC (Wang et al., 2023; Watson et al., 2015).

The treatment landscape for mHSPC has undergone significant transformation, with the introduction of novel therapies such as ADT + docetaxel or second-generation antiandrogens demonstrating substantial improvements in survival outcomes (Lam et al., 2024; Hamilou et al., 2018; Kyriakopoulos et al., 2018). The LATITUDE trial was the first to validate the efficacy of the abiraterone acetate plus prednisone (AAP) + ADT doublet regimen (Fizazi et al., 2019a). Studies have shown that this combination leads to a decline in prostate-specific antigen (PSA) levels (Sadaghiani et al., 2022), with the extent of PSA reduction serving as a key early indicator of long-term prognosis (Sadaghiani et al., 2021; Chowdhury et al., 2023). The emergence of the PEACE-1 trial further demonstrated that the triplet regimen of AAP + ADT + docetaxel improves both overall survival (OS) and radiographic progression-free survival (rPFS) in mHSPC patients (Fizazi et al., 2022), establishing this triplet regimen as a standard treatment. With the continuous development of second-generation antiandrogens, darolutamide (DARO) has gained attention. The ARASENS trial highlighted the significant OS benefits of the DARO + ADT + docetaxel triplet regimen (Smith et al., 2022). More recently, the ARANOTE study confirmed the safety and efficacy of the DARO + ADT doublet regimen (Sa et al., 2024), ushering in a new era of dual therapy for mHSPC.

Both DARO and AAP triplet regimen are associated with significantly increased adverse event rates and higher treatment costs (Lee, 2023; Hoeh et al., 2023; Shore et al., 2022). In real-world settings, patients often exhibit reluctance toward chemotherapy due to its side effects, influencing their treatment choices (Jansen et al., 2022; Aparicio et al., 2021). Concerns regarding drug accessibility, tolerability, safety, drug-drug interactions, and health economics have led many mHSPC patients to opt for doublet regimen only (Leith et al., 2022; Freedland et al., 2021; Raval et al., 2024; Schiff, 2022).

Although the efficacy of the doublet and triplet regimen of DARO and AAP has been studied in a controlled environment, real - world data can more comprehensively demonstrate their performance in clinical applications. Patients are resistant to chemotherapy drugs and intolerant to side effects. Meanwhile, there is a lack of head - to - head comparisons of the efficacy and safety of the DARO and AAP doublet regimen in the real world. This study focuses on the real - world setting and compares the efficacy and safety of the DARO + ADT and AAP + ADT doublet regimen for the treatment of mHSPC, with the time to mCRPC as the primary endpoint. The aim is to provide evidence for the clinical application of the DARO + ADT doublet regimen, strive to improve the efficacy and safety, and reduce the economic burden on patients.

## 2 Methods

### 2.1 Patients and treatment

This study retrospectively evaluated the efficacy and safety of DARO + ADT *versus* AAP + ADT in the treatment of mHSPC patients in the real world. The clinical data of mHSPC patients who were treated with DARO + ADT or AAP + ADT and visited Sichuan Provincial People’s Hospital from January 2022 to June 2024 were retrospectively analyzed, and the follow-up lasted until December 2024. In the treatment regimens, the dose of DARO was 600 mg administered orally twice daily in combination with ADT in the DARO + ADT regimen. In contrast, the dose of abiraterone acetate was 1,000 mg administered orally once daily, and the dose of prednisone was 5 mg administered orally twice daily in the AAP + ADT regimen.

The inclusion criteria for patients were as follows: ① Pathologically or cytologically confirmed prostate adenocarcinoma; ② Performance status score of 0–1 according to the Eastern Cooperative Oncology Group (ECOG); ③ Received DARO + ADT or AAP + ADT treatment for at least 1 month and had relatively complete follow-up data; ④ Testosterone was at the castration level during the treatment process (testosterone <50 ng/mL or <1.7 nmol/L). The exclusion criteria were: ① Received docetaxel treatment previously or during the follow-up; ② Had severe underlying diseases that were poorly controlled; ③ Received palliative radiotherapy, palliative surgery or particle implantation simultaneously; ④ Had a history of other primary malignancies, except for patients with *in situ* carcinoma who had no evidence of disease for 5 years or more and did not require treatment.

### 2.2 Follow-up observation data and endpoints

We collected the clinical data of patients from the hospital information electronic medical record system (HIS), including: age, Gleason score of the puncture pathology, ECOG score, baseline testosterone, PSA levels (before treatment, 1 month, 3 months, 6 months, 9 months, 12 months after treatment; time to reach PSA50 (defined as the proportion of patients with a 50% decrease in PSA from the baseline value after treatment), PSA90 (defined as the proportion of patients with a 90% decrease in PSA from the baseline value after treatment), PSA <2 ng/mL, PSA <0.02 ng/mL, and PSA <0.008); previous treatment history and drug-related adverse events (AE).

The primary endpoint was the time to mCRPC, and the secondary endpoints included OS, rPFS, time to PSA progression, time to subsequent prostate cancer therapy.

### 2.3 Data analysis

SPSS 26.0 software was used to conduct statistical analysis of the relevant data. The Kolmogorov-Smirnov method was used to test the normality of the measurement data. The measurement data with non-normal distribution were represented by M (P25, P75) and analyzed by the Mann-Whitney U test. The count data were represented by the number of cases (%) and analyzed by the χ^2^ test or Fisher’s exact probability method. The Kaplan-Meier estimation and COX regression model were used to analyze the primary and secondary endpoints. A *P* value less than 0.05 indicated that the difference was statistically significant.

## 3 Results

### 3.1 Baseline data

A total of 308 patients were included in the study, and 178 of them met the inclusion criteria. Among them, 96 patients used DARO and 82 patients used AAP. None of the included patients had received docetaxel chemotherapy. The demographic and baseline characteristics of the patients were well balanced between the two groups (*P* > 0.05) (Table 1). The age distribution was consistent in both groups. The median age in the DARO group was 74 years (53–96 years), and the median age in the AAP group was 75 years (48–75 years). In the DARO group, 75 patients (78.1%) had a Gleason score of ≥8; in the AAP group, 67 patients (81.7%) had a Gleason score of ≥8. In terms of the ECOG score, 61 patients (63.5%) in the DARO group had an ECOG score of 0, and 54 patients (65.9%) in the AAP group had an ECOG score of 0; 35 patients (36.5%) in the DARO group had an ECOG score of 1, and 28 patients (34.1%) in the AAP group had an ECOG score of 1.

**TABLE 1:** Baseline data on 178 patients.

Characteristics	DARO values	AAP values	*P* Value
Age (years), median [range]	74 [53–96]	75 [48–95]	0.307
<70, n (%)	25 (26.0)	21 (25.6)	
≥70, n (%)	71 (74.0)	61 (74.4)	
Gleason score, median [IQR]	8 [8–9]	8 [8–9]	0.06
≥8, n (%)	75 (78.1)	67 (81.7)	
<8, n (%)	21 (21.9)	15 (18.3)	
ECOG, median [range]	0 [0–1]	0 [0–1]	0.748
0, n (%)	61 (63.5)	54 (65.9)	
1, n (%)	35 (36.5)	28 (34.1)	
Prior treatment, n (%)	50 (52.1)	45 (54.9)	0.709
Prior treatment regimen			
ADT alone, n/N (%)	13/50 (26.0)	10/45 (22.2)	
ADT + BICA, n/N (%)	35/50 (70.0)	35/45 (77.8)	
ADT + AA, n/N (%)	2/50 (4.0)		
Baseline PSA (ng/mL), median [IQR]	22.97 [7.44–87.17]	22.91 [5.45–66.67]	0.442
≥20, n (%)	52 (54.2)	44 (53.7)	
≥100, n (%)	21 (21.9)	8 (9.8)	
Testosterone (ng/mL), median [IQR]	0.39 [0.09–9.49]	0.45 [0.09–0.91]	0.489
Comorbidities, n (%)	39 (40.6)	27 (32.9)	0.289

DARO, darolutamide; AAP, abiraterone acetate plus prednisone; IQR, interquartile range; ECOG, eastern cooperative oncology group; ADT, androgen deprivation therapy; BICA, bicalutamide; AA, abiraterone acetate; PSA, prostate-specific antigen.

Among all 96 patients who received DARO, 50 patients (52.1%) had a history of previous treatment, and 46 patients (47.9%) received DARO as the first-line treatment. Among 82 patients who received AA, 45 patients (54.9%) had a history of previous treatment, and 37 patients (45.1%) received AA as the first-line treatment.

### 3.2 Primary end point

**FIGURE 1:**
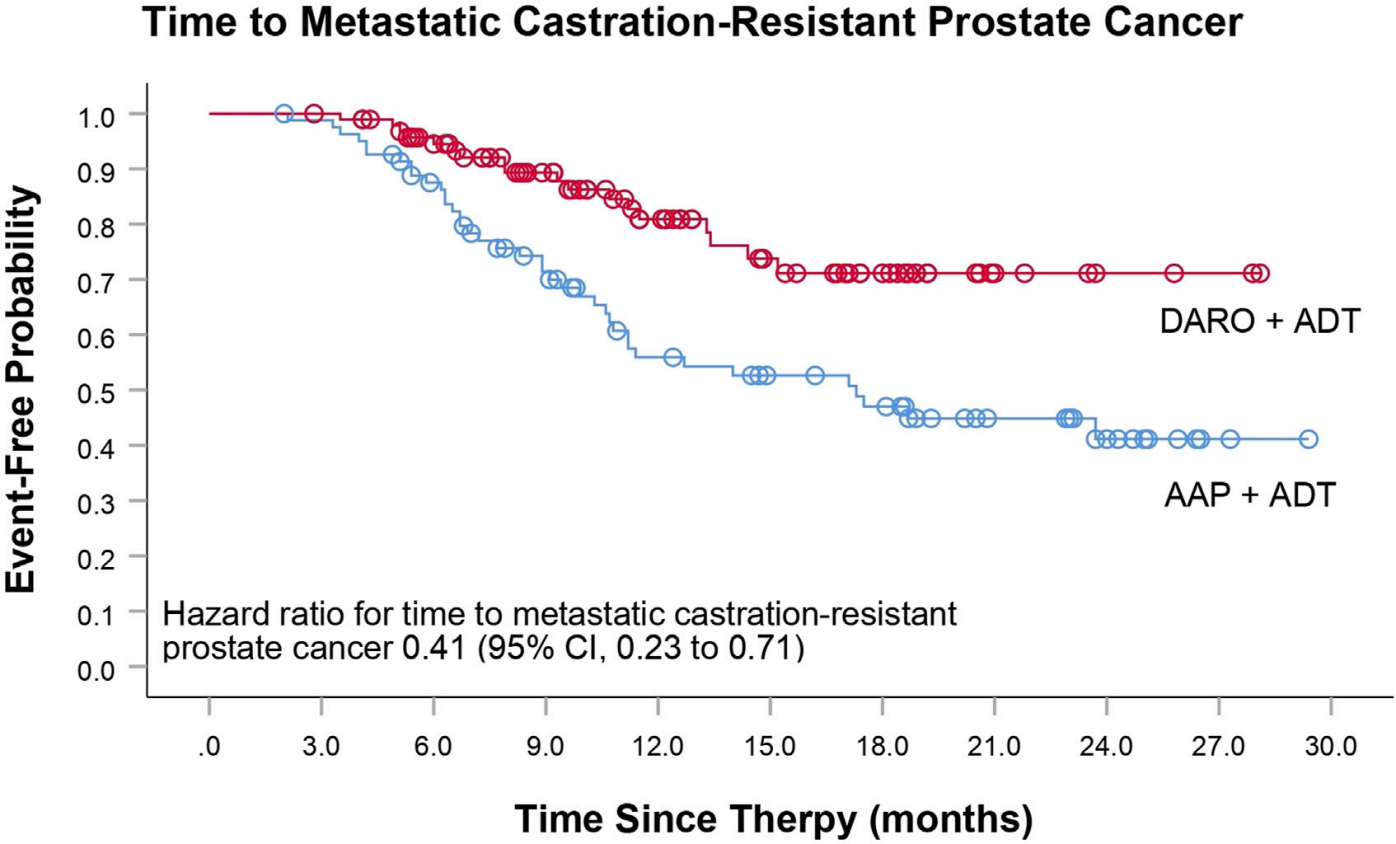
Time to metastatic castration-prostate cancer Kaplan-Meier estimates.

The primary endpoint of this study was the time to mCRPC. The analysis of time to mCRPC demonstrated that the proportion of patients progressing in the DARO group (18/92; 18.8%) was significantly lower than that in the AAP group (39/82; 47.6%). DARO significantly extended the time to progression to mCRPC, with a 59% reduction in the risk of progression to mCRPC compared to the AAP group [HR, 0.41 (95% CI, 0.23 to 0.71); *P <* 0.005] (Table 2; Figure 1). The median time to progression was not reached in the DARO group, whereas it was 17.3 months in the AAP group.

**TABLE 2:** Time to event end points.

End points	DARO + ADT (n = 96)		AAP + ADT (n = 82)		Hazard Ratio (95% CI)	*P* value
	Median, months	Events, (%)	Median, months	Events, (%)		
Time to mCRPC	NR	18 (18.8)	17.3	39 (47.6)	0.41 (0.23,0.71)	0.002
Time to PSA progression	NR	15 (15.6)	22.3	34 (41.5)	0.42 (0.23,0.78)	0.006
OS	NR	4 (4.2)	NR	18 (22.0)	0.31 (0.10,0.93)	0.037
rPFS	NR	6 (6.3)	NR	27 (32.9)	0.21 (0.09,0.51)	0.001
Time to pain progression	NR	7 (7.3)	NR	18 (22.0)	0.37 (0.16,0.90)	0.028
Time to subsequent prostate cancer therapy	NR	3 (3.1)	NR	14 (17.1)	0.23 (0.07,0.82)	0.023

DARO, darolutamide; ADT, androgen-deprivation therapy; AAP, abiraterone acetate plus prednisone; mCRPC, metastatic castration-resistant prostate cancer; PSA, prostate-specific antigen; OS, overall survival; rPFS, radiological progression-free survival; NR, not reached. A hazard ratio and 95% CI, are based on Cox regression model.

### 3.3 Secondary efficacy end points

**FIGURE 2:**
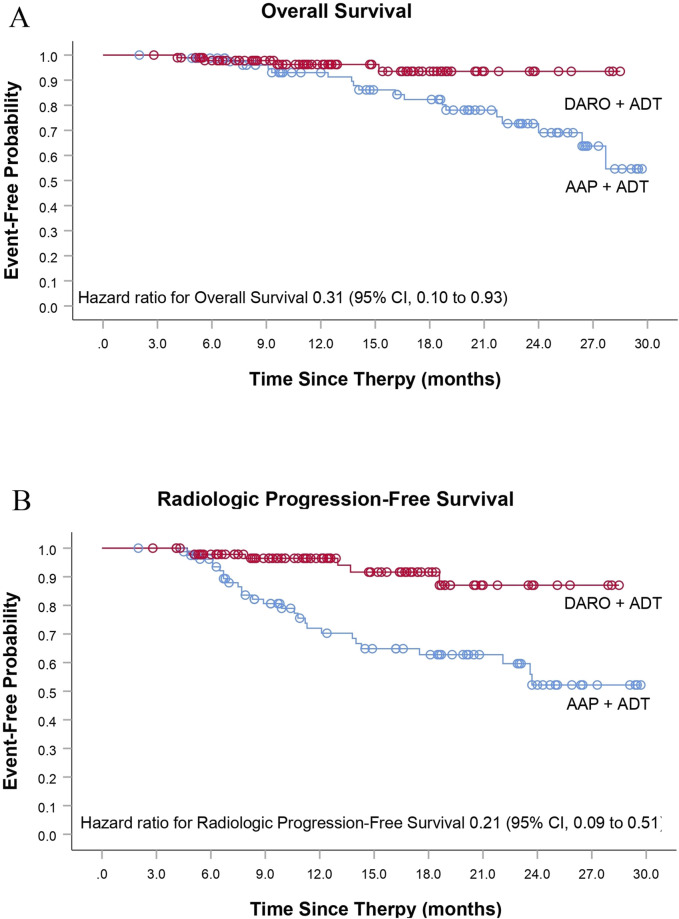
Additional secondary time-to-event end points. **(A)** Overall Survival and **(B)** Radiologic Progression-Free Survival.

The DARO group had obvious benefits in all secondary endpoints compared with the AAP group. Among the patients who experienced PSA progression during the follow-up, the proportion of patients in the DARO group (15/96; 15.6%) was less than that in the AAP group (34/82; 41.5%). The risk of PSA progression in the DARO group was 58% lower than that in the AAP group [HR, 0.42 (95% CI, 0.23 to 0.78); *P <* 0.05]. The median time in the DARO group was not reached, while the median time in the AAP group was 22.3 months. The hazard ratios of OS and rPFS in the DARO group were 69% and 79% lower than those in the AAP group respectively [HR, 0.31 (95% CI, 0.10 to 0.93); *P <* 0.05 for OS; HR, 0.21 (95% CI, 0.09 to 0.51); *P <* 0.005 for rPFS] (Table 2; Figure 2). Similarly, compared with the AAP group, the time to pain progression [HR, 0.37 (95% CI, 0.16 to 0.90); *P <* 0.05] and the time to subsequent prostate cancer therapy [HR, 0.23 (95% CI, 0.07 to 0.82); *P <* 0.05] were both delayed in the DARO group.

### 3.4 PSA response rate

This study retrospectively analyzed changes in PSA levels following treatment. Compared to baseline, the median PSA reduction rates in the DARO group at 1, 3, 6, 9, and 12 months were 95.1%, 96.4%, 92.8%, 84.2%, and 61.7%, respectively, while those in the AAP group were 34.7%, 71.2%, 70.5%, 50.0%, and 45.9%, respectively. The median follow-up times [IQR] for the DARO and AAP groups were 12.0 [7.9–17.6] months and 17.4 [9.3–23.8] months, respectively. The Mann-Whitney U test indicated no significant difference in baseline PSA levels between the two groups (*P* > 0.05), confirming comparability.

In the DARO group (n = 96), 95, 92, 85, 66, and 51 patients had PSA follow-up data at 1, 3, 6, 9, and 12 months, respectively. The PSA50 response rates were 94.7%, 96.7%, 95.3%, 95.5%, and 94.1%, while the PSA90 response rates were 72.6%, 84.8%, 91.8%, 84.8%, and 78.4%, respectively. In the AAP group (n = 82), 81, 77, 71, 60, and 58 patients had PSA follow-up data at the corresponding time points. The PSA50 response rates were 50.6%, 76.6%, 74.6%, 71.7%, and 70.7%, while the PSA90 response rates were 24.7%, 49.4%, 60.6%, 58.3%, and 56.9%, respectively. Pearson’s chi-square test revealed that the DARO group had significantly higher PSA50 and PSA90 response rates at all time points compared to the AAP group (*P <* 0.05).

Furthermore, the proportions of patients achieving PSA <2 ng/mL, <0.2 ng/mL, and <0.008 ng/mL in the DARO group were 99%, 86.5%, and 74.0%, respectively, compared to 86.6%, 70.7%, and 51.2% in the AAP group. The differences between the two groups were statistically significant (*P <* 0.05), with the DARO group demonstrating superior outcomes (Table 3).

**TABLE 3:** Number and proportion of DARO and AAP groups with PSA at the corresponding value.

PSA response	DARO	AAP	Pearson chi-square	*P* Value
1-month PSA response, n/N (%)				
PSA50	90/95 (94.7)	41/81 (50.6)	44.720	<0.001
PSA90	69/95 (72.6)	20/81 (24.7)	40.199	<0.001
3-month PSA response, n/N (%)				
PSA50	89/92 (96.7)	59/77 (76.6)	15.587	<0.001
PSA90	78/92 (84.8)	38/77 (49.4)	24.447	<0.001
6-month PSA response, n/N (%)				
PSA50	81/85 (95.3)	53/71 (74.6)	13.613	<0.001
PSA90	78/85 (91.8)	43/71 (60.6)	21.642	<0.001
9-month PSA response, n/N (%)				
PSA50	63/66 (95.5)	43/60 (71.7)	13.318	<0.001
PSA90	56/66 (84.8)	35/60 (58.3)	11.014	0.001
12-month PSA response, n/N (%)				
PSA50	48/51 (94.1)	41/58 (70.7)	9.942	0.002
PSA90	40/51 (78.4)	33/58 (56.9)	5.690	0.017
Rate of PSA <2 ng/mL, n (%)	95 (99.0)	71 (86.6)	10.769	0.001
Rate of PSA <0.2 ng/mL, n (%)	83 (86.5)	58 (70.7)	6.643	0.010
Rate of PSA <0.008 ng/mL, n (%)	71 (74.0)	42 (51.2)	9.864	0.002

DARO, darolutamide; AAP, abiraterone acetate plus prednisone; PSA, prostate-specific antigen; PSA50, prostate-specific antigen reduction by 50% or more; PSA90, prostate-specific antigen reduction by 90% or more.

### 3.5 Drug-related adverse reactions

According to the previous medical records of patients in this study, the incidence of adverse reactions during the follow-up was similar between the two groups. In the DARO group, it was 22/96 (22.9%), and in the AAP group, it was 27/82 (32.9%). The adverse reactions with relatively high incidences in the DARO group included gastrointestinal reactions, rash, constipation, and abnormal liver function, etc.; the adverse reactions with relatively high incidences in the AAP group included abnormal liver function, hypertension, fatigue, and gastrointestinal reactions, etc. No drug-related adverse reactions above grade 3 were reported in either group.

## 4 Discussion

This study evaluated the efficacy and safety of DARO + ADT in patients with mHSPC. In the assessment of the primary endpoint, we compared the time to mCRPC. The results showed that the time to mCRPC in the DARO group was significantly prolonged. This finding indicates that DARO has a favorable effect in delaying disease progression compared to AAP. This contradicts the meta-analysis by Lin Wang et al., who concluded that abiraterone acetate had the lowest risk of metastasis and death (Wang et al., 2022). However *Rana* McKay concluded that in the real-world, patients receiving DARO have better outcomes (Rana et al., 2025), which is consistent with this study. In addition, the COX regression analysis between the DARO group and the AAP group also showed a significant difference, suggesting that the DARO doublet regimen has an advantage in reducing the risk of mHSPC progressing to mCRPC. DARO significantly improved OS with consistent safety in phase III trials involving patients with non - metastatic castration resistant prostate cancer (nmCRPC) and mHSPC (in combination with ADT and Docetaxel) (Smith et al., 2022; Fizazi et al., 2019b). These findings highlight that in real - world data, the efficacy of the DARO doublet regimen may be superior to that of the AAP doublet regimen.

In the assessment of secondary efficacy endpoints, the comparison of the time to PSA progression is equally important. The study found that the DARO group showed a significant advantage in the time to PSA progression, which may be related to its stronger anti-tumor activity (Sung et al., 2021). The analysis results of OS and rPFS also support this view, indicating that patients in the DARO group can maintain a progression-free state for a longer time after treatment (Gillessen et al., 2023). The comparison of the time to pain progression provides a basis for evaluating the safety of treatment, showing that the DARO group has an advantage in reducing bone-related symptoms (Sagaram and Rao, 2021). The comparison of the time to subsequent prostate cancer therapy reflects the impact of different treatment regimens on patients' subsequent treatment choices, suggesting that clinicians need to comprehensively consider patients’ long-term management needs when formulating treatment plans (Wei et al., 2025).

The results of this study showed that compared with the AAP group, the PSA level in the DARO group was significantly reduced. In the comparison of PSA response rates, we observed changes in PSA levels at different time points, especially the differences between the DARO group and the AAP group. This finding is consistent with previous research results, indicating that DARO treatment may significantly reduce PSA levels in the early stage (Jafari et al., 2024; Tombal et al., 2022). At the same time, the comparison of PSA50 and PSA90 remission rates showed that the remission rates in the DARO group were significantly higher than those in the AAP group, which further supports the potential advantages of DARO in treatment. For the achievement of PSA <2 ng/mL, <0.02 ng/mL, and <0.008 ng/mL, our statistical analysis results also showed significant differences. These results are consistent with previous studies that emphasized the importance of early PSA reduction in improving the prognosis and survival rate of mHSPC patients (Liu et al., 2024; Myint et al., 2022; Wenzel et al., 2024), and also indicate that the DARO doublet regimen is more effective than AAP in reducing PSA levels and can provide a new treatment option for clinical practice.

This study aims to compare the efficacy of DARO + ADT *versus* AAP + ADT in the treatment of mHSPC in a real-world setting, addressing a significant gap in clinical evidence. The results demonstrated that the DARO group significantly outperformed the AAP group in both primary and secondary endpoints, including PSA changes and time to mCRPC (*P* < 0.05), highlighting the beneficial role of the DARO + ADT combination in mHSPC management, offering the possibility of choosing the DARO + ADT doublet in patients who are unable to choose the DARO triple therapy and supporting its doublet potential as a first-line treatment option. However, these results will only concern patients ineligible for the triplet. And the retrospective design of the study may introduce bias, and the limited follow-up duration precludes a comprehensive assessment of long-term efficacy and safety.

Despite Laila A. Gharzai’s assertion that no endpoint has been validated as a surrogate endpoint for OS, that caution is essential when employing rPFS as a surrogate endpoint in clinical trial design, and that follow-up metrics must be optimized for enhanced assessment in future studies (Gharzai et al., 2023), the use of rPFS as the primary endpoint is not unprecedented. In numerous large-scale randomized controlled trials (RCTs), rPFS has also been used as a primary endpoint in some studies, although OS is often selected as the primary endpoint. In the present study, the relatively low number of patient deaths observed during the follow-up period, coupled with the unique characteristics of mHSPC led to the selection of rPFS as the primary endpoint. Future research should involve larger-scale, longer-term, multicenter, prospective studies with appropriate follow-up endpoints selected to further validate these findings and explore their implications for personalized treatment strategies.

## 5 Conclusion

This real-world study demonstrates that DARO + ADT significantly improves clinical outcomes compared to AAP + ADT in the treatment of mHSPC. DARO + ADT delayed the time to mCRPC and showed superior PSA reduction rates, higher PSA50 and PSA90 response rates, and better secondary endpoints, including OS and rPFS. Both regimens exhibited comparable safety profiles, with no grade 3 or higher adverse events. These findings support DARO + ADT as a promising first-line treatment for mHSPC, though larger, prospective studies are needed to confirm long-term efficacy and safety.

## Data Availability

The raw data supporting the conclusions of this article will be made available by the authors, without undue reservation.
